# Scarlet Red in Gastric and Duodenal Ulcer

**Published:** 1913-11

**Authors:** 


Scarlet Red in Gastric and Duodenal Ulcer. Julius Friedenwald and T. F. Letiz of Baltimore, *Monthly Cyc. and Med. Bull.,* June, 1913, report 37 cases, either ambulant or previously treated in vain by the Leube rest or Lenhartz dietetic method, cured or markedly benefited. The dosage was about 3-4 grams a day for 3-8 weeks, the drug being best administered in half gram konseals.

